# Differentiating between dengue fever and malaria using hematological parameters in endemic areas of Thailand

**DOI:** 10.1186/s40249-017-0238-x

**Published:** 2017-03-02

**Authors:** Manas Kotepui, Bhukdee PhunPhuech, Nuoil Phiwklam, Kwuntida Uthaisar

**Affiliations:** 1 Medical Technology Program, School of Allied Health Sciences, Walailak University, Nakhon Si Thammarat, 80161 Thailand; 2 Medical Technology Laboratory, Phop Phra Hospital, PhopPhra District, Tak Province 63160 Thailand

**Keywords:** Dengue, Malaria, Hematological parameters, Markers

## Abstract

**Background:**

Dengue fever (DF) and malaria are the two major public health concerns in tropical countries such as Thailand. Early differentiation between dengue and malaria could help clinicians to identify patients who should be closely monitored for signs of dengue hemorrhagic fever or severe malaria. This study aims to build knowledge on diagnostic markers that are used to discriminate between the infections, which frequently occur in malaria-endemic areas, such as the ones in Thailand.

**Methods:**

A retrospective study was conducted in Phop Phra Hospital, a hospital located in the Thailand-Burma border area, a malaria-endemic area, between 2013 and 2015. In brief, data on 336 patients infected with malaria were compared to data on 347 patients infected with DF.

**Results:**

White blood cells, neutrophil, monocyte, eosinophil, neutrophil-lymphocyte ratio, and monocyte-lymphocyte ratio were significantly lower in patients with DF compared to patients with malaria (*P* < 0.0001). In contrast, red blood cells, hemoglobin, hematocrit, mean corpuscular volume, mean corpuscular hemoglobin, and mean corpuscular hemoglobin concentration were significantly higher in patients with DF as compared to patients with malaria (*P* < 0.0001). A decision tree model revealed that using neutrophils, lymphocyte, MCHC, and gender was guided to discriminate between dengue and malaria infection.

**Conclusion:**

This study concluded that several hematological parameters were different for diagnosing DF and malaria. A decision tree model revealed that using neutrophils, lymphocyte, MCHC, and gender was guided to discriminate patients with dengue and malaria infection. In addition, using these markers will thus lead to early detection, diagnosis, and prompt treatment of these tropical diseases.

**Electronic supplementary material:**

The online version of this article (doi:10.1186/s40249-017-0238-x) contains supplementary material, which is available to authorized users.

## Multilingual abstracts

Please see Additional file 1 for translations of the abstract into the five official working languages of the United Nations.

## Background

Dengue fever (DF) and malaria are the two most common arthropod-borne diseases in tropical countries, especially in Southeast Asian regions, where their endemic areas greatly overlap [1, 2]. The clinical presentations of both diseases are usually unspecific and are difficult to distinguish between each other. Currently in 2015, the World Health Organization (WHO) reports that *P. vivax* malaria makes up 75% of all malaria cases, and most cases of *P. vivax* malaria occurred in the WHO South-East Asia Region (74%) [1]. In 2009, 23 229 cases of malaria and 19 deaths were reported in Thailand, with *P. vivax* being responsible for half of the total cases [2, 3].

Dengue fever is caused by the dengue virus (DENV) in one of the four serotypes: DENV-1, DENV-2, DENV-3, and DENV-4 [4]. According to estimates of the WHO, about 50 million patients are infected with DF annually worldwide and 2.5 billion people live in risk areas [5]. Dengue is endemic in Thailand, with 86 653 cases and nine deaths reported in 2014 [6]. Dengue infection can range from a nonspecific febrile illness, as in DF, to a more severe illness with bleeding tendency, thrombocytopenia, and plasma leakage (dengue hemorrhagic fever, DHF) [7]. At presentation, DF and other febrile illnesses share similar clinical characteristics, including headache, myalgia, and rash. However, clinical characteristics of DHF, such as bleeding and plasma leakage, are seen at a later stage of the febrile phase after the third or fourth day of fever. Patients are classified as having DHF, according to the WHO guidelines, based on all of the following four signs: fever, thrombocytopenia (platelet count < 100 000/μL), bleeding (positive tourniquet test or spontaneous bleeding), and plasma leakage (evidence of pleural effusion, ascites or ≥ 20% hemoconcentration) [8].

In Thailand, DF and malaria coexist, and are both prevalent. Early differentiation between dengue and malaria could help clinicians to identify patients who should be closely monitored for signs of DHF or severe malaria. Differences in clinical and laboratory features between DF and malaria have been reported, however, published studies vary on information pertaining to the duration of symptoms and demographic of patients.

Phop Phra Hospital is located in Tak Province, a territory located in the western part of Thailand, which shares a long border with Kayin State, Myanmar. It is part of the Thailand-Myanmar border area and is the most common destination for people from Myanmar who migrate to Thailand for agricultural work. During malaria endemics, large numbers of patients with fever and other febrile illnesses are admitted weekly to emergency. There is risk of misdiagnosing malaria cases as DF cases in a malaria-endemic area, as the two diseases share similar clinical and laboratory characteristics. In addition, it may be impossible to both examine for dengue and malaria for all patients exhibiting similar signs and symptoms.

The objective of this study was to identify clinical and laboratory features that can differentiate between patients infected with dengue and those infected with malaria, as well as to build knowledge about diagnostic markers used to discriminate DF from malaria, which both frequently occur in malaria-endemic areas.

## Methods

A retrospective case-control study designed to differentiate between dengue and malaria was performed between January 2013 and December 2015 at the Medical Technology Laboratory of Phop Phra Hospital, Tak Province, Thailand.

In brief, the patients infected only with DF and those infected only with malaria were exhaustively identified based on the date of microbiological diagnosis. The diagnosis of malaria relied on the identification of sporozoa on thick/thin blood film stained with Giemsa and the identification of parasites under a light microscope by medical laboratorists. Thick and thin blood films were prepared by collecting venous blood in an Ethylenediaminetetraacetic acid (EDTA) tube and staining it with diluted Giemsa (1:10). One thousand red blood cells (RBCs) on the thin blood film and 200 white blood cells (WBCs) on the thick smear were examined before classifying a case as negative. EDTA blood were also analysed to determine the complete blood counts (CBCs) using a BC-5200 Hematology Analyzer (Mindray, Nanshan, Shenzhen, China). The analyzer provided data on RBC count, hemoglobin (Hb), hematocrit (Hct), platelet count, WBC count, neutrophil, monocyte, lymphocyte and eosinophil counts, mean corpuscular volume (MCV), mean corpuscular hemoglobin (MCH), and mean corpuscular hemoglobin concentration (MCHC).

Dengue can be diagnosed in laboratories using different methods. Nonstructural protein 1 (NS1) antigen detection was performed from day 0 to day 5, and indirect diagnosis based on the detection of specific anti-dengue immunoglobulin M (IgM) or immunoglobulin G (IgG) antibodies in patients’ sera after day 3 [9]. At least one positive test was taken to mean a positive dengue infection. Data were retrieved anonymously from patients and from computerized medical records, and included socioepidemiologic characteristics, previous medical history, and biological results.

A normal distribution of continuous data were determined using the Kolmogorov-Smirnov test. Continuous variables were categorized following laboratory or usual cut-off values. For example, WBCs < 4 000/μL, neutrophils < 2 800/μL, lymphocytes < 800/μL, monocytes < 80/μL, eosinophils < 40/μL, basophils < 16/μL, RBCs < 4 × 10^6^/μL, Hb < 11 g/dL, and platelets < 100 000/μL were considered lower than cut-off values. Hematocrit > 40%, MCV > 80 fL, MCH > 25 pg/cell, MCHC > 33 g/dL, and red cell distribution width (RDW) >13% were considered higher than cut-off values. Continuous variables were compared using the Mann–Whitney *U* test. Categorical variables were analysed using the Fisher’s exact test or chi-square test. Statistical significance was set at *P*-value less than 0.05. Univariate logistic regression model was performed to confirm that associations between variables were independent of age, gender, and nationality. Variables with *P*-values of less than 0.05 in univariate logistic regression analyses were entered into a multivariate logistic regression model. Conditional multivariate backward stepwise logistic regression was then performed to calculate the adjusted odds ratios (*OR*s) and 95% confidence intervals (95%*CI*s). All significant variables in the univariate and multivariate logistic model were then used to determine a diagnostic value of DF versus malaria. The diagnostic accuracy of the hematological parameters was measured by calculating their sensitivity, specificity, predictive values, and *OR*s and 95%*CI*s.

A decision tree model (RapidMiner studio basic v7.1, RapidMiner, Inc., USA) was built to analyse parameters related to dengue and malaria infection. The goal of the analysis was to identify the most important parameters using the demographic data of the study population and hematological parameters.

## Results

### Demographic data of the study population

The 683 patients who were investigated in this study had a median age of 19 years (interquartile range, IQR: 10–31 in the dengue group, 11–34 in the malaria group). No significant differences relating to age were observed between the two groups (*P* = 0.299). There were more women (51.6%) in the dengue group and more men (61.3%) in the malaria group. A significant difference relating to gender between the two groups was observed (*P* = 0.001, *OR* 95%*CI* = 0.59 [0.44–0.80]). The large majority of people in both groups were Thai (dengue group = 74.1%; malaria group = 52.1%). A significant difference relating to nationality between the two groups was observed (*P* < 0.0001, *OR* 95%*CI* = 2.6 [1.91–3.62]) (see Table 1).

**Table 1:** General characteristics of the study participants

	Dengue *N* = 347	*Plasmodium* sp*.* malaria *N* = 336	*P*-value (*OR*, 95%*CI*)
Demographic			
Age, median (IQR)	19 (10–31)	19 (11–34)	0.299*
Male/female, *n* (%)	168 (48.4)/179 (51.6)	206 (61.3)/130 (38.7)	0.001** (0.59, 0.44–0.80)
Thai/non-Thai, *n* (%)	257 (74.1)/90 (25.9)	175 (52.1)/161 (47.9)	<0.0001** (2.6, 1.91–3.62)

*Comparison of the two groups was done using the Mann-Whitney *U* Test

**Comparison of the two groups was done using the chi-square test

### Microbiological results

Out of the study cohort, 336 patients were infected with malaria. Of those, 72.9% were infected with *P. vivax* and 21.1% were infected with *P. falciparum.* Meanwhile, 347 patients were infected with dengue. Of these, 20.7% were positive with NS1 antigen but IgM or IgG test was not tested, 17.6% were positive with dengue IgG antibody but NS1 antigen was not tested, and 12.4% were positive just with IgM antibody. Co-infection of dengue and malaria was not found. The microbiological results are shown in Table 2.

**Table 2:** Laboratory diagnoses of dengue and malaria

	Frequency	Percentage (%)
*Plasmodium* sp*.*		
*P. vivax*	245	72.9
*P. falciparum*	91	21.1
Total	336	100
Dengue (NS1, IgM, IgG)		
+, NA, NA	72	20.7
NA, −, +	61	17.6
NA, +, −	43	12.4
+, −, −	36	10.4
-, +, +	35	10.1
NA, +, +	31	8.9
+, +, +	28	8.1
-, −, +	21	6.1
+, +, −	13	3.7
+, −, +	7	2
Total	347	100

*NA* Not applicable

### Hematological values of the study population

The following parameters were significantly lower in patients with DF as compared to patients with malaria (*P*-value less than 0.0001): WBCs, neutrophil, monocyte, eosinophil, NL ratio, and monocyte-lymphocyte ratio (ML ratio). The following parameters were significantly higher patients with DF as compared to patients with malaria (*P* < 0.0001): RBCs, Hb, Hct, MCV, MCH, and MCHC (*P* < 0.009). The following parameters were not significantly difference in patients with DF compared to patients with malaria (*P* > 0.05): lymphocyte, basophil, RDW, and platelet count (see Table 3).

**Table 3:** Hematological values of the study population

Variable	Dengue Median (IQR)	*Plasmodium* sp*.* Median (IQR)	*P*-value*
WBCs (×10^3^/μL)	3.79 (2.64–5.51)	5.95 (4.81–7.51)	<0.0001
Neutrophil (×10^3^/μL)	2.19 (1.41–3.20)	4.12 (2.99–5.35)	<0.0001
Lymphocyte (×10^3^/μL)	1.05 (0.69–1.63)	1.05 (0.67–1.64)	0.607
Monocyte (×10^3^/μL)	0.20 (0.11–0.33)	0.29 (0.14–0.54)	<0.0001
Eosinophil (×10^3^/μL)	0.05 (0.03–0.09)	0.11 (0.06–0.19)	<0.0001
Basophil (×10^3^/μL)	0.06 (0.03–0.12)	0.06 (0.04–0.09)	0.258
NL ratio	1.184 (1.15–1.84)	3.94 (2.17–7.23)	<0.0001
ML ratio	0.17 (0.09–0.27)	0.27 (0.13–0.50)	<0.0001
RBC (×10^6^/μL)	5.02 (4.63–5.53)	4.66 (4.22–5.07)	<0.0001
Hb (g/dL)	13.6 (12.4–15.0)	12.3 (10.8–13.4)	<0.0001
HcT (%)	40 (36–44)	36 (32–40)	<0.0001
MCV (fL)	81.4 (75–85.5)	78.4 (74.0–83.4)	<0.0001
MCH (pg/cell)	27.8 (25.3–29.3)	26.5 (24.8–28.3)	<0.0001
MCHC (g/dL)	34 (33.4–34.7)	33.9 (33–34.5)	0.009
RDW (%)	12.3 (11.9–13.0)	12.4 (11.8–13.1)	0.866
Platelet (×10^3^/μL)	76 (45–101)	80.5 (52.3–119)	0.053

*Comparison of the two groups was done using the Mann-Whitney *U* Test

### Diagnostic values of hematological parameters

Most of the hematological parameters had average sensitivity and specificity to differentiating between dengue and malaria. However, a MCH higher than 25 pg/cell had the highest sensitivity (78%) and an eosinophil count less than 40 cells/μL had the highest specificity (94%) to differentiate between patients with DF and those with malaria. Hematological parameters that had both good sensitivity and specificity were: neutrophil count less than 2 800 cells/μL (sensitivity 66%, specificity 80%) and a NL ratio of less than 2.8 (sensitivity 72%, specificity 65%). Hematological parameters that had good ORs were WBC count less than 4 000 cells/μL (*OR* = 7.52, 95%*CI* = 5.12–11.03), neutrophil count less than 2 800 cells/μL (*OR* = 7.65, 95%*CI* = 5.41–10.82), and eosinophil count less than 40 cells/μL (*OR* = 7.63, 95%*CI* = 4.65–12.51) (see Table 4).

**Table 4:** Sensitivity, specificity, predictive value, and OR of hematological parameters for differentiating between dengue and malaria

Variable	Sensitivity* (95%*CI*)	Specificity* (95%*CI*)	PPV* (95%*CI*)	NPV* (95%*CI*)	*P*-value**	OR (95%*CI*)	PLR	NLR
WBCs < 4 000/μL	52.5 (47.1–57.8)	87.2 (83.2–90.6)	80.9 (75.1–85.8)	64 (59.4–68.4)	<0.0001	7.52 (5.12–11.03)	4.10 (3.10–5.50)	0.55 (0.48–0.61)
Neutrophils < 2 800/μL	66 (75.1–83.9)	79.8 (75.1–83.9)	77.1 (71.9–81.8)	69.4 (64.6–74.0)	<0.0001	7.65 (5.41–10.82)	3.26 (2.6–4.1)	0.43 (0.36–0.5)
Lymphocytes < 800/μL	33.7 (28.8–39.0)	65.2 (59.8–70.3)	50.8 (47.0–54.6)	48.8 (44.1–53.5)	0.761	0.95 (0.69–1.31)	0.97 (0.79–1.19)	1.02 (0.91–1.13)
Monocytes < 80/μL	15.3 (11.7–19.5)	85.1 (80.9–88.8)	51.5 (41.4–61.4)	49.3 (45.2–53.5)	0.89	1.03 (0.68–1.57)	1.03 (0.72–1.47)	1 (0.93–1.06)
Eosinophils < 40/μL	33.7 (28.8–39.0)	93.8 (90.6–96.1)	84.8 (77.7–90.3)	57.8 (53.5–62.0)	<0.0001	7.63 (4.65–12.51)	5.39 (3.48–8.37)	0.71 (0.65–0.77)
Basophils < 16/μL	12.7 (9.37–16.7)	80.4 (75.7–84.5)	40.0 (30.8–49.8)	47.1 (43.0–51.3)	0.013	0.594 (0.39–0.90)	0.65 (0.45–0.92)	1.09 (1.02–1.16)
NL ratio < 2.8	71.5 (66.4–76.2)	64.9 (59.5–70.0)	67.8 (62.7–72.5)	68.8 (63.4–73.8)	<0.0001	4.63 (3.35–6.39)	2.04 (1.73–2.39)	0.44 (0.37–0.53)
ML ratio < 0.25	72.3 (67.3–77.0)	53.6 (48.1–59.0)	61.7 (56.8–66.4)	65.2 (59.3–70.8)	<0.0001	1.77 (1.48–2.12)	1.56 (1.37–1.78)	0.52 (0.42–0.63)
RBC < 4 × 10^6^/μL	6.34 (4.02–9.44)	87.6 (84.2–90.6)	29.0 (19.1–40.5)	54.0 (50.3–57.8)	<0.0001	0.3 (0.21–59)	0.51 (0.32–0.82)	1.07 (1.02–1.12)
Hb < 11 g/dL	7.49 (4.95–10.8)	74.1 (69.1–78.7)	23.0 (15.6–31.9)	43.7 (39.6–47.9)	<0.0001	0.23 (0.15–0.37)	0.29 (0.19–0.44)	1.25 (1.16–1.34)
Hct > 40%	45.8 (40.5–51.2)	81.9 (77.3–85.8)	72.3 (65.9–78.1)	59.4 (54.8–63.9)	<0.0001	3.81 (2.69–5.41)	2.52 (1.96–3.25)	0.66 (0.59–0.74)
MCV > 80 fL	58.2 (52.8–63.5)	59.5 (54.1–64.8)	59.8 (54.3–65.0)	58.0 (52.6–63.2)	<0.0001	2.05 (1.51–2.78)	1.44 (1.23–1.68)	0.70 (0.60–0.82)
MCH > 25 pg/cell	78.1 (73.4–82.3)	26.2 (21.6–31.2)	52.2 (47.8–56.6)	53.7 (45.7–61.5)	0.19	1.27 (0.89–1.79)	1.06 (0.97–1.15)	0.84 (0.64–1.09)
MCHC > 33 g/dL	52.7 (47.3–58.1)	53.0 (47.5–58.4)	53.7 (48.2–59.1)	52.1 (46.6–57.5)	0.135	1.26 (0.93–1.70)	1.12 (0.96–1.13)	0.89 (0.77–1.04)
RDW > 13%	25.4 (20.9–30.3)	71.7 (66.6–76.5)	48.1 (40.7–55.6)	48.2 (43.7–52.7)	0.390	0.86 (0.61–1.21)	0.9 (0.70–1.15)	1.04 (0.95–1.14)
Platelets +< 100 000/μL	73.8 (68.8–78.3)	33.6 (28.6–39.0)	53.4 (48.9–58.0)	55.4 (48.3–62.3)	0.034	1.43 (1.23–1.98)	1.11 (1.01–1.23)	0.78 (0.62–0.98)

*PLR* positive likelihood ratio, *NLR* negative likelihood ratio

*Percent, **P-value determined using the chi-square test

### Regression analysis of parameters and dengue/malaria infection

After adjusting for age, gender, and nationality in the univariate regression model, the association between hematological parameters (WBCs, neutrophil, monocyte, eosinophil, NL ratio, ML ratio, RBCs, Hb, Hct, MCH) and dengue infection were not confound (*P* < 0.05) (see Table 5). After adjusting for age, gender, and nationality of patients in the multivariate regression model, the association between hematological parameters (WBCs, neutrophil, monocyte, eosinophil, MCV, MCH, and MCHC) and dengue infection were not confound (*P* < 0.05) (see Table 6).

**Table 5:** Univariate analysis of dengue/malaria infection and hematological parameters adjusted by age, gender, and nationality

Parameters	Dengue/malaria infection	*P*-value	Exp (B)
WBCs (×10^3^/μL)	0.302	<0.0001	1.35
Neutrophil (×10^3^/μL)	0.598	<0.0001	1.82
Monocyte (×10^3^/μL)	1.54	<0.0001	4.67
Eosinophil (×10^3^/μL)	6.65	<0.0001	772.95
NL ratio	0.209	<0.0001	1.23
ML ratio	2.116	<0.0001	8.29
RBCs (×10^6^/μL)	−0.943	<0.0001	0.389
Hb (g/dL)	−0.458	<0.0001	0.632
HcT (%)	−0.156	<0.0001	0.855
MCV (fL)	−0.01	0.239	0.99
MCH (pg/cell)	−0.099	<0.0001	0.906
MCHC (g/dL)	−0.028	0.556	0.973

**Table 6:** Multivariate analysis of dengue/malaria infection and hematological parameters adjusted by age, gender, and nationality

Parameters	Dengue/malaria infection	*P*-value	Exp (B)
WBCs (×10^3^/μL)	−0.523	0.001	0.592
Neutrophil (×10^3^/μL)	1.086	<0.0001	2.96
Monocyte (×10^3^/μL)	1.98	0.037	7.28
Eosinophil (×10^3^/μL)	4.86	<0.0001	130.3
NL ratio	−0.004	0.910	0.995
ML ratio	−0.002	0.997	0.997
RBCs (×10^6^/μL)	−0.031	0.659	0.968
Hb (g/dL)	−0.054	0.815	0.947
HcT (%)	−0.160	0.054	0.851
MCV (fL)	0.231	0.001	1.26
MCH (pg/cell)	−0.638	0.001	0.528
MCHC (g/dL)	0.199	0.007	1.22
Constant	−3.119	0.249	0.044

### The decision tree model

**Fig. 1:**
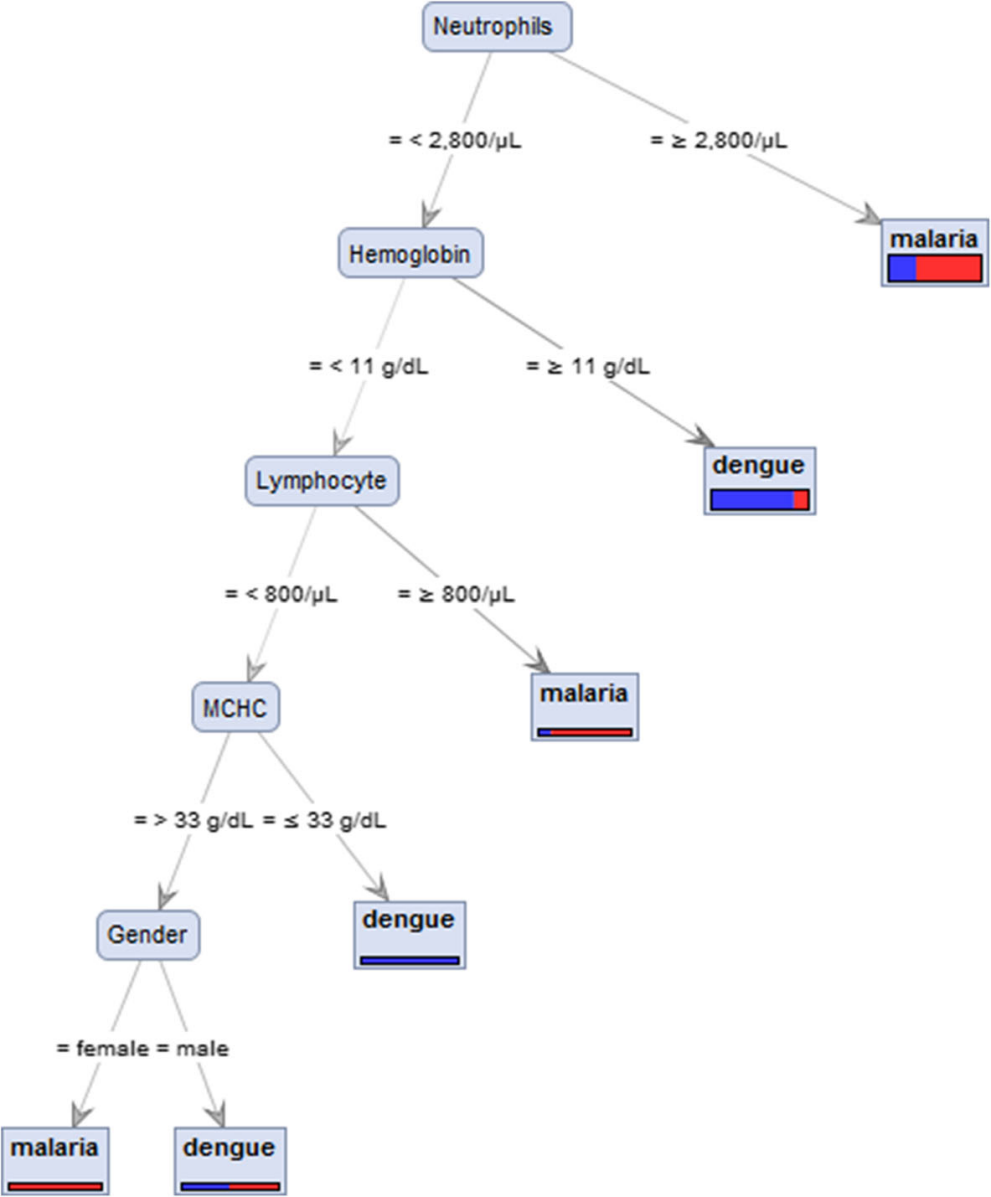
Decision tree used to differentiate between patients with malaria and patients with dengue

The decision tree model generated from the dataset is shown in Fig. 1. All individuals were divided into six subgroups (six nodes) from root node (neutrophils) to nodes (hemoglobin, lymphocyte, MCHC, and gender) through different branches (multiple arrows) and finally to leaf (dengue or malaria). The possibility to discriminate dengue infection from malaria infection was varied from 5% to 100%. For example, using neutrophils < 2 800/μL, Hb < 11 g/dL, lymphocyte < 800/μL, and MCHC ≤ 33 g/dL could help to increase specificity for discriminating patients with DF. In addition, using neutrophils < 2 800/μL, Hb < 11 g/dL, lymphocyte < 800/μL, and MCHC ≥ 33 g/dL, and female patients could help to increase specificity for discriminating patients with patients with malaria infection.

## Discussion

The present study confirmed that sociodemographic characteristics differ between patients with DF and those with malaria.

Firstly, patients with DF tended to be younger than patients with malaria, however, no significant difference between the two infections and age were observed (mean age DF = 22.5, malaria = 24.1 years old). This was in concordance to another study, which indicated that patients with DF tended to be younger (a quarter of the study participants being below 15 years of age) [10]. It has been reported that the incidence of malaria in endemic areas decreases as people grow older, which suggests that older age contributes to immunity against malaria [11]. One possible explanation is age-related differences in immune function leading to differences in the balance between protective and harmful host immune responses to *P. falciparum* [12]. Previous studies have also suggested that the risk of developing severe dengue is greater in DENV-infected children (<15 years) than in adults [13, 14].

Secondly, significantly more male patients were infected with malaria than with dengue in this study, which a previous study also correlated [10]. This may due to adult males reporting at least one journey into the forest or working in the forest, which may have made them more susceptible to getting bitten by a malaria-borne mosquito.

Third, although over a third of the patients in this study were from Burma, significantly more Thai patients had DF than malaria. This could be explained by the fact that dengue is transmitted in urban areas and not in the forest. Most of the patients with malaria were infected with *P. vivax* (73%), which was similar to a report from the WHO [1]. Nowadays, drug-resistant *P. vivax* malaria cases are increasing. It is usually assumed that *P. falciparum* is the most likely *Plasmodium* species to cause severe malaria, however, there is growing evidence that *P. vivax* can also lead to severe complications [15].

In the present study, hematological presentation was more severe and abnormalities were less frequent in patients with DF than in those with malaria. This might be partly due to the fact that patients with dengue presented early after the onset of symptoms. Red blood cells, Hb, Hct, MCV, MCH, and MCHC were significantly lower in patients with malaria. This may due to the parasite’s primary target being RBCs, resulting in an accelerated removal of both parasitized and non-parasitized RBCs by the destruction of RBCs and bone marrow dysfunction [16, 17]. One report indicated that a high level of malaria parasitemia was associated with high RBC destruction [18]. Anemia (Hb level < 11 g/dL) was frequently found in malaria positive patients, especially patients infected with the *P. falciparum* strain [3].

Hemoconcentration and raised Hct are common in patients with DHF [19]. A high Hct value is the initial abnormality occurring in DHF due to plasma leakage. Increase in Hct concentration by more than 20% of the baseline Hct is an important diagnostic criterion [20]. This study found that the median Hct in patients with dengue was 40%, which was in the normal range. However, diagnosis data of whether the patient had DHF could not be obtained in this study, such as a positive tourniquet test or spontaneous bleeding, and plasma leakage [8]. This study found that a higher level of MCV, MCH, and MCHC. However, association of these parameters and pathogenesis of malaria infection remain inconclusive. However, a previous study showed that those parameters were higher in patients infected with malaria as compared to the non-malaria infected group [3].

White blood cells, neutrophil, monocyte, eosinophil, NL ratio, and ML ratio were significantly lower in patients with DF than in those with malaria. Leukopenia is common in DF patients and a useful diagnostic marker [7]. The total decrease in leukocyte count when a patient has dengue is mainly due to a decrease in granulocytes such as neutrophils [21]. Neutropenia in dengue infections has also been reported, but less frequently [22, 23]. However, it was more frequently observed in this study (neutrophils were lower in 66% of patients with dengue). A previous study indicated that severe neutropenia was not associated with an increased risk of secondary bacterial infections [24], but may be due to leukocytes having been infected with DENV. Infected cells (dendritic cells and Langerhans cells) then migrate from the site of infection to the lymph nodes, where monocytes and macrophages are recruited, thus becoming targets of infection [25]. The suppression of WBC production in bone marrow by dengue virus may be a possible mechanism for lower leukocytes [26]. Presence of atypical lymphocytes with activated lymphocytes is reported consistently along with CBC parameters [21], but was not applicable in this study. Both the NL ratio and ML ratio in this study were significant lower in patients with dengue than those with malaria due to a prominent increase in the percentage and total number of lymphocytes, and an increase in the percentage and number of atypical lymphocytes [22]. It has been reported that for lower eosinophil counts in patients with dengue, eosinophil concentrations fell and during convalescence, the eosinophil concentrations rose to a normal range in response to inflammation during the acute phase of the infection [27].

Previous studies have aimed to establish criteria to differentiate between malaria attacks and DF. A study performed in southern India to compare malaria to other infectious causes of fever found that normal leukocyte counts, moderate to severe thrombocytopenia, renal failure, splenomegaly, and hyperbilirubinemia with elevated serum transaminases were associated with malaria. However, rash, overt bleeding tendency, normal to low leukocyte counts, moderate to severe thrombocytopenia, and significantly elevated hepatic transaminases were associated with DF [28]. A previous study also found that patients with DF had a high packed cell volume (PCV), whereas patients with malaria had a low PCV. In addition, patients with malaria had the lowest platelet count compared to patients with DF [29].

A low platelet count is a classic feature of both infections. This study confirmed that both patients with DF and malaria had low platelet counts, however, no significant differences between the two groups were observed. In many tropical regions, fever with thrombocytopenia is assumed to be malaria [3], and fever with thrombocytopenia and a negative blood smear is thought to be DF [10]. In the present study, thrombocytopenia (<100 000/μL) was less common in patients with DF than in those with malaria (DF = 74%, malaria = 66%). A study comparing the biological features of DF, malaria, and leptospirosis in India showed similar results, observing that thrombocytopenia was a predictor of malaria [29]. A previous study also suggested that platelet counts were lower in patients with malaria compared to patients with DF, especially at first presentation [30]. This could be explained by the fact that platelet counts in patients with DF are typically the lowest 3–6 days from onset, when fever is about to decrease [31]. The cause of thrombocytopenia in DF is unknown, but decreased production of platelets and increased destruction of platelets in DHF have previously been described [21, 32]. Other explanations have been a direct infection of the megakaryocytes by virus leading to increased destruction of the platelets or increased antibodies directed against the platelets [33]. Coagulopathy, which was not assessed in this study, is also common in most patients with DF. Previous studies have reported that prolonging of activated partial thromboplastin time is quite common, whereas prothrombin time was normal in the majority of patients [34–36].

In this study, co-infection of dengue and malaria was not found. Previous reports rationalized that the main habitat of malaria vectors is the forest [37], whereas the main habitat of dengue vectors is the city [38]; that immunity to either in its endemic area might prevent infections [39]; and that co-infection can be easily forgotten [40]. Although hematological parameters of co-infected patients were not available in this study, a previous study suggested that low HcT (<36%) and deep thrombocytopenia (<50G/L) were significantly found in patients with a co-infection as compared to patients infected just with malaria. In addition, low Hb (<12 g/dL), low HcT (<36%), and deep thrombocytopenia (<50G/L) were significantly found in patients with co-infections as compared to patients infected just with dengue [41].

This study had certain limitations such as the fact that the amount of days that patients had fever before they were admitted to hospital could not be determined from the hospital’s database. In addition, this study lacks of previous medical histories that may confound the analysis such as Hb diseases, bacteria or virus infection. Further study is needed to validate whether the proposed parameters were likely to be altered during the febrile stage of the illnesses and whether the proposed indicators can differentiate between dengue and malaria.

## Conclusions

The findings of this study suggest that several clinical and laboratory measures could potentially distinguish between patients with DF and those with malaria. The sensitivity, specificity, positive predictive value (PLV), negative predictive value (NPV), and diagnostic accuracy for all hematological parameters were determined. A tree decision model revealed that using neutrophils, lymphocyte, MCHC, and gender was guided to discriminate of patients with malaria and DF. This model may be used to identify patients with dengue and those with malaria, but it needs to be used in the early stages of the illnesses in order for it to be useful for reducing unnecessary medication and time of hospitalizations.

## Additional file

Additional file 1: Multilingual abstracts in the five official working languages of the United Nations. (PDF 675 kb)

## Abbreviations

CBC: Complete blood count
CI: Confidence interval
DENV: Dengue virus
DF: Dengue fever
DHF: Dengue hemorrhagic fever
Hb: Hemoglobin
Hct: Hematocrit
IgG: Immunoglobulin G
IgM: Immunoglobulin M
IQR: Interquartile range
MCH: Mean corpuscular hemoglobin
MCHC: Mean corpuscular hemoglobin concentration
MCV: Mean corpuscular volume
ML: Monocyte-lymphocyte ratio
NL: Neutrophil-lymphocyte
NPV: Negative predictive value
NS1: Nonstructural protein 1
OR: Odds ratio
PCV: Packed cell volume
PPV: Positive predictive value
RBC: Red blood cell
RDW: Red cell distribution width
WBC: White blood cell
WHO: World Health Organization

## References

[1] World Health Organization (2015). World Malaria Report.

[2] Bureau of Epidemiology. Malaria. Department of Disease Control Ministry of Public Health, Nonthaburi. 2015. http://www.boe.moph.go.th/boedb/surdata/annualdata/d3052.pdf. Accessed 22 Feb 2016.

[3] Kotepui M, Phunphuech B, Phiwklam N, Chupeerach C, Duangmano S (2014). Effect of malarial infection on haematological parameters in population near Thailand-Myanmar border. Malar J.

[4] Anderson CR, Downs WG, Hill AE (1956). Isolation of dengue virus from a human being in Trinidad. Science.

[5] World Health Organization (2009). Dengue: guidelines for diagnosis, treatment, prevention and control.

[6] Bureau of Epidemiology (2016). Dengue fever.

[7] Potts JA, Rothman AL (2008). Clinical and laboratory features that distinguish dengue from other febrile illnesses in endemic populations. Trop Med Int Health.

[8] World Health Organization (1997). Dengue haemorrhagic fever: diagnosis, treatment, prevention, and control.

[9] Dussart P, Petit L, Labeau B, Bremand L, Leduc A, Moua D (2008). Evaluation of two new commercial tests for the diagnosis of acute dengue virus infection using NS1 antigen detection in human serum. PLoS Negl Trop Dis.

[10] Epelboin L, Boulle C, Ouar-Epelboin S, Hanf M, Dussart P, Djossou F (2013). Discriminating malaria from dengue fever in endemic areas: clinical and biological criteria, prognostic score and utility of the C-reactive protein: a retrospective matched-pair study in French Guiana. PLoS Negl Trop Dis.

[11] Malik AM, Zaffar N, Ali N, Khan R (2010). Haematological findings and endemicity of malaria in Gadap region. J Coll Physicians Surg Pak.

[12] Baird JK (1995). Host age as a determinant of naturally acquired immunity to Plasmodium falciparum. Parasitol Today.

[13] Carlos CC, Oishi K, Cinco MT, Mapua CA, Inoue S, Cruz DJ (2005). Comparison of clinical features and hematologic abnormalities between dengue fever and dengue hemorrhagic fever among children in the Philippines. Am J Trop Med Hyg.

[14] Guzman MG, Kouri G, Bravo J, Valdes L, Vazquez S, Halstead SB (2002). Effect of age on outcome of secondary dengue 2 infections. Int J Infect Dis.

[15] Abdallah TM, Abdeen MT, Ahmed IS, Hamdan HZ, Magzoub M, Adam I (2013). Severe Plasmodium falciparum and Plasmodium vivax malaria among adults at Kassala Hospital, eastern Sudan. Malar J.

[16] Price RN, Simpson JA, Nosten F, Luxemburger C, Hkirjaroen L, ter Kuile F (2001). Factors contributing to anemia after uncomplicated falciparum malaria. Am J Trop Med Hyg.

[17] Kitua AY, Smith TA, Alonso PL, Urassa H, Masanja H, Kimario J (1997). The role of low level Plasmodium falciparum parasitaemia in anaemia among infants living in an area of intense and perennial transmission. Trop Med Int Health.

[18] Kotepui M, Piwkham D, PhunPhuech B, Phiwklam N, Chupeerach C, Duangmano S (2015). Effects of malaria parasite density on blood cell parameters. PLoS One.

[19] Itoda I, Masuda G, Suganuma A, Imamura A, Ajisawa A, Yamada K (2006). Clinical features of 62 imported cases of dengue fever in Japan. Am J Trop Med Hyg.

[20] World Health Organization, No.22,. Monograph on Dengue/Dengue Hemorrhagic Fever. 1993.

[21] Kalayanarooj S, Vaughn DW, Nimmannitya S, Green S, Suntayakorn S, Kunentrasai N (1997). Early clinical and laboratory indicators of acute dengue illness. J Infect Dis.

[22] Ali N, Usman M, Syed N, Khurshid M (2007). Haemorrhagic manifestations and utility of haematological parameters in dengue fever: a tertiary care centre experience at Karachi. Scand J Infect Dis.

[23] Chadwick D, Arch B, Wilder-Smith A, Paton N (2006). Distinguishing dengue fever from other infections on the basis of simple clinical and laboratory features: application of logistic regression analysis. J Clin Virol.

[24] Thein TL, Lye DC, Leo YS, Wong JG, Hao Y, Wilder-Smith A (2014). Severe neutropenia in dengue patients: prevalence and significance. Am J Trop Med Hyg.

[25] Durbin AP, Vargas MJ, Wanionek K, Hammond SN, Gordon A, Rocha C (2008). Phenotyping of peripheral blood mononuclear cells during acute dengue illness demonstrates infection and increased activation of monocytes in severe cases compared to classic dengue fever. Virology.

[26] La Russa VF, Innis BL (1995). Mechanisms of dengue virus-induced bone marrow suppression. Baillieres Clin Haematol.

[27] Beeson PBBD (1977). The eosinophils. Major problems in internal medicine.

[28] Chrispal A, Boorugu H, Gopinath KG, Chandy S, Prakash JA, Thomas EM (2010). Acute undifferentiated febrile illness in adult hospitalized patients: the disease spectrum and diagnostic predictors - an experience from a tertiary care hospital in South India. Trop Doct.

[29] Shah I, Katira B (2007). Clinical and laboratory profile of dengue, leptospirosis and malaria in children: a study from Mumbai. Arch Dis Child.

[30] Kutsuna S, Hayakawa K, Kato Y, Fujiya Y, Mawatari M, Takeshita N (2015). Comparison of clinical characteristics and laboratory findings of malaria, dengue, and enteric fever in returning travelers: 8-year experience at a referral center in Tokyo, Japan. J Infect Chemother.

[31] World Health Organization. Dengue: guidelines for diagnosis, treatment, prevention and control 2009. 23762963

[32] Cardier JE, Marino E, Romano E, Taylor P, Liprandi F, Bosch N (2005). Proinflammatory factors present in sera from patients with acute dengue infection induce activation and apoptosis of human microvascular endothelial cells: possible role of TNF-alpha in endothelial cell damage in dengue. Cytokine.

[33] Ostronoff M, Ostronoff F, Florencio R, Florencio M, Domingues MC, Calixto R (2003). Serious thrombocytopenia due to dengue hemorrhagic fever treated with high dosages of immunoglobulin. Clin Infect Dis.

[34] Jameel T, Mehmood K, Mujtaba G, Choudhry N, Afzal N, Paul RF (2012). Changing haematological parameters in dengue viral infections. J Ayub Med Coll Abbottabad.

[35] Ali N, Nadeem A, Anwar M, Tariq WU, Chotani RA (2006). Dengue fever in malaria endemic areas. J Coll Physicians Surg Pak.

[36] Wills BA, Oragui EE, Stephens AC, Daramola OA, Dung NM, Loan HT (2002). Coagulation abnormalities in dengue hemorrhagic Fever: serial investigations in 167 Vietnamese children with Dengue shock syndrome. Clin Infect Dis.

[37] Obsomer V, Defourny P, Coosemans M (2007). The Anopheles dirus complex: spatial distribution and environmental drivers. Malar J.

[38] Cox J, Grillet ME, Ramos OM, Amador M, Barrera R (2007). Habitat segregation of dengue vectors along an urban environmental gradient. Am J Trop Med Hyg.

[39] Jordan W (1985). New approaches to vaccine development. Proceedings of a meeting organized by the World Health Organization. Book review. Int Health News.

[40] Ward DI (2006). A case of fatal Plasmodium falciparum malaria complicated by acute dengue fever in East Timor. Am J Trop Med Hyg.

[41] Epelboin L, Hanf M, Dussart P, Ouar-Epelboin S, Djossou F, Nacher M (2012). Is dengue and malaria co-infection more severe than single infections? A retrospective matched-pair study in French Guiana. Malar J.

