# Supplementary material for: Differentiating between dengue fever and malaria using hematological parameters in endemic areas of Thailand
Source: Infect Dis Poverty. 2017 Mar 2;6:27. doi: 10.1186/s40249-017-0238-x (PMC5333447; doi:10.1186/s40249-017-0238-x)
Supplement: Additional file 1: — Multilingual abstracts in the five official working languages of the United Nations. (PDF 675 kb) [file 40249_2017_238_MOESM1_ESM.pdf]

Translation of the abstract into the five official working languages of the United Nations

التمييز بين حُمى الضنك والملاريا باستخدام معايير الدم في المناطق الموطونة في تايلاند

مناس كوتيبوي، وبوكدي فويش، ونويل فيوكلام، وكونتيدا أوثيسار

ملخّص:
معلومات أساسية: تُشكّل حُمىّ الضنك النزفية، والملاريا هاجسيْن رئيسييْن للصحة العامة في البلدان المدارية مثل تايلاند. ومن شأن التمييز مبكراً بين حُمّى الضنك النزفية، والملاريا أن يُعين الأطباء السريريين على التعرف على المرضى الذين يتعيّن مراقبتهم عن كثب بسبب علامات حمي الضنك النزفية، أو الملاريا الخبيثة. والهدف من هذه الدراسة هو زيادة المعرفة بشأن العلامات التشخيصية التي تُستخدم للتمييز بين هاتين الإصابتين اللتين تحدثان بصورة متكررة في مناطق مستوطنة بالملاريا مثل تايلاند.
الطُرُق: أُجريت دراسةٌ سابقة، بين عاميْ 2013 و2015، في مستشفى فوب فرا، الذي يقع في المنطقة الحدودية بين تايلاند وبورما، وهي منطقة مستوطنة بالملاريا. وإيجازاً، فقد جرت مقارنة بيانات 336 مريض مصاب بالملاريا مع بيانات 347 مريض مصاب بحمّي الضنك النزفية.
النتائج: كانت خلايا الدم البيضاء، والخلايا المتعادلة، والخلايا وحيدة النواة، والخلايا الحامضية، والخلايا اللمفاوية، ونسبة الخلايا المتعادلة إلى الخلايا اللمفاوية، ونسبة الخلايا وحيدة النواة إلى الخلايا اللمفاوية لدى مَرْضى حُمّي الضنك النزفية أقل كثيراً من مَرْضَى الملاريا (قيمة الرجحان المحسوب < 0,0001). وعلى العكس من ذلك، كانت خلايا الدم الحمراء، والهيموجلوبين (خضاب الدم)، والهيماتوكريت (حجم الخلايا المكدّسة)، ومتوسط حجم الكريات، ومتوسط جسيمات الهبموجلوبين، ومتوسط تركيز الهيموجلوبين في كريات الدم الحمراء لدى مرضى حمى الضنك النزفية أعلى كثيراً من مَرْضى الملاريا (قيمة الرجحان المحسوب < 0,0001). وقد كشف نموذج شجرة القرار أنه جرى الاهتداء باستخدام الخلايا المتعادلة، والخلايا اللمفاوية، ومتوسط تركيز الهيموجلوبين، وجنس المريض لتمييز المصابين بحمى الضنك النزفية، عن المصابين بالملاريا.
الخلاصة: خلُصت الدراسة إلى أن العديد من معايير الدم المستخدمة في تشخيص حمى الضنك النزفية كانت تختلف عن تلك المستخدمة للملاريا. وقد كشف نموذج شجرة القرار أنه جرى الاهتداء باستخدام الخلايا المتعادلة، والخلايا اللمفاوية، ومتوسط تركيز الهيموجلوبين، وجنس المريض للتمييز بين المصابين بحمى الضنك النزفية، والمصابين بالملاريا. فضلاً عن ذلك، فإن استخدام هذه العلامات سيؤدي بالتالي إلى الكشف المبكّر لهذه الأمراض المداريّة والبدء فوراً في علاجها.

Translated from English version into Arabic by Hassan Adam, through

TRANSLATORS
WITHOUT BORDERS

在泰国流行区依据血液学参数区分登革热和疟疾

Manas Kotepui, Bhukdee PhunPhuech, Nuoil Phiwklam, Kwuntida Uthaisar

摘要
引言：在热带国家，如泰国登革热（DF）和疟疾是两个主要备受关注的公共卫生问题。登革热和疟疾的早期区分，可以帮助临床医生甄别哪些患者应该接受登革出血热或者恶性疟等相关症状的严密监控。本研究拟在疟疾流行区如泰国确定区分上述两种感染的诊断标志物。
方法：在泰缅边境疟疾流行地区的 Phop Phra 医院开展了一项回顾性研究，对该院 2013-2015 年，336 例疟疾患者与 347 例 DF 患者的相关数据进行比较。

结果：与疟疾患者相比，DF 患者的白细胞、中性粒细胞、单核细胞、嗜酸性粒细胞、中性粒细胞-淋巴细胞比率和单核细胞-淋巴细胞比率均显著降低（$P < 0.0001$）。相反的，与疟疾患者相比，DF 患者的红细胞、血红蛋白、血细胞比容、平均红细胞体积、平均红细胞血红蛋白和平均红细胞血红蛋白浓度（MCHC）显著升高（$P < 0.0001$）。决策树模型揭示，中性粒细胞、淋巴细胞、MCHC 可区分登革热和疟疾。
结论：本研究发现有几个血液参数可用于诊断 DF 和疟疾。决策树模型显示，中性粒细胞、淋巴细胞和 MCHC 可用于区分登革热和疟疾患者。此外，使用这些诊断标志物可对这两种疾病进行早期检测、诊断和及时治疗。

Translated from English version into Chinese by Lu-Lu Haung, edited by Pin Yang

CHINESE CENTER FOR DISEASE CONTROL AND PREVENTION
NATIONAL INSTITUTE OF PARASITIC DISEASES

Différencier la Dengue et le Paludisme par l'utilisation de paramètres hématologiques, dans les zones endémiques de Thaïlande.

Manas Kotepui, Bhukdee PhunPhuech, Nuoil Phiwklam, Kwuntida Uthaisar

Sommaire

Contexte: la Dengue et le Paludisme sont deux problèmes de santé publique majeurs dans des pays tropicaux comme la Thaïlande. Une distinction précoce entre la Dengue et le Paludisme pourrait permettre aux praticiens de déterminer quels patients devraient être étroitement surveillés en cas de Dengue hémorragique ou de Paludisme sévère. Cette étude a pour but d'accroître les connaissances sur les marqueurs diagnostiques utilisés pour distinguer ces deux infections, qui sévissent souvent dans les zones endémiques de paludisme, comme par exemple dans celles de Thaïlande.
Méthodes: Une étude rétrospective a été dirigée à l'hôpital de Phop Phra, un hôpital situé dans la région frontalière de Thaïlande-Burma, une zone endémique du Paludisme, entre 2013 et 2015. En résumé, des données relatives à 336 patients atteints de Paludisme ont été comparées à des données relatives à 347 patients atteints de la Dengue.
Résultats: les taux de globules blancs, de neutrophile, de monocyte, d'éosinophile, de neutrophile-lymphocyte, et les taux de monocyte-lymphocyte étaient significativement plus bas chez les patients atteints de la Dengue comparé aux patients infectés par le Paludisme (P-value <0.0001). Par contre, les globules rouges, l'hémoglobine, les hématocrites, le volume globulaire moyen, l'hémoglobine globulaire moyenne, et la concentration d'hémoglobine globulaire moyenne étaient significativement plus élevés chez le patients atteints de la Dengue, comparés aux patients atteints de Paludisme (P-value <0.0001). Un modèle d'arbre décisionnel a révélé que l'utilisation de neutrophiles, de lymphocytes, MCHC, et genre a été guidée pour différencier l'infection de la Dengue de celle du Paludisme.
Conclusion: Cette étude a conclu que plusieurs paramètres hématologiques étaient différents pour diagnostiquer la Dengue et le Paludisme. Un modèle d'arbre décisionnel a révélé que l'utilisation de neutrophiles, de lymphocytes, MCHC, et genre a été guidée pour différencier les patients infectés par la Dengue de ceux infectés par le Paludisme. En outre, l'utilisation de ces marqueurs mènera ainsi à une détection précoce, un diagnostic, et un traitement prompt de ces maladies tropicales.

Translated from English version into French by Ode Laforge, through

Установление различий между лихорадкой денге и малярией при помощи гематологических параметров в эндемических областях Таиланда

Манас Котепуи (Manas Kotepui), Бхукди Фунфуек (Bhukdee PhunPhuech), Нуоил Фивклам (Nuoil Phiwklam), Квунтида Уфайсар (Kwuntida Uthaisar)

Аннотация

Краткое описание. Лихорадка денге (ЛД) и малярия являются двумя основными болезнями, угрожающими населению в тропических странах, таких как Таиланд. Раннее установление различий между денге и малярией поможет врачам выявить пациентов, которым необходимо наблюдение за появлением признаков геморрагической лихорадки денге или тяжёлой малярии. Данное исследование направлено на накопление знаний о диагностических признаках, используемых для распознавания этих болезней, которые часто встречаются в эндемичных для малярии областях, таких как в Таиланде.

Методы. В период между 2013 и 2015 годами было проведено ретроспективное исследование в госпитале Фоп-Фра (Phop Phra), расположенном в эндемичной для малярии области на таиландско-бирманской границе. Данные 336 больных малярией были сравнены с данными 347 больных, заражённых ЛД.

Результаты. Количество лейкоцитов, нейтрофилов, моноцитов и эозинофилов, а также соотношения нейтрофилов/лимфоцитов и моноцитов/лимфоцитов у больных ЛД были значительно ниже по сравнению с больными малярией ($P < 0{,}0001$). В то же время количество эритроцитов, уровни гемоглобина и гематокрита, средняя гематокритная величина, средний эритроцитарный объём и среднеклеточная концентрация гемоглобина у больных ЛД были значительно выше по сравнению с больными малярией ($P < 0{,}0001$). Дерево принятия решений показало, что использование таких параметров, как нейтрофилы, лимфоциты, средняя концентрация клеточного гемоглобина в эритроцитах и пол, позволяет различить денге и малярию.

Выводы. Данное исследование пришло к выводу, что при диагностировании ЛД и малярии отличаются несколько гематологических параметров. Дерево принятия решений показало, что использование таких параметров, как нейтрофилы, лимфоциты, средняя концентрация клеточного гемоглобина в эритроцитах и пол, позволяет различить денге и малярию. Использование упомянутых признаков также приводит к раннему выявлению, диагнозу и незамедлительному лечению этих тропических болезней.

Translated from English version into Russian by Natalia Potashnik, through

Diferenciación entre fiebre del dengue y malaria mediante parámetros hematológicos en áreas endémicas de Tailandia

Manas Kotepui, Bhukdee PhunPhuech, Nuoil Phiwklam, Kwuntida Uthaisar

Resumen

Información de referencia: La fiebre del dengue y la malaria constituyen los dos aspectos más preocupantes para la salud pública en países tropicales como Tailandia. La diferenciación en fase temprana entre dengue y malaria podría ayudar a los médicos a identificar a los pacientes a los que se debería hacer un seguimiento minucioso en busca de indicios de fiebre hemorrágica del dengue o de malaria grave. Este estudio tiene como objetivo aumentar el conocimiento sobre los marcadores diagnósticos que se utilizan para distinguir entre las infecciones que se producen a menudo en áreas endémicas de malaria, como las situadas en Tailandia.

Métodos: Entre 2013 y 2015 se realizó un estudio retrospectivo en el Hospital Phop Phra, que se encuentra en la zona limítrofe entre Tailandia y Birmania, que es endémica de malaria. Se compararon los datos de 336 pacientes infectados con malaria con los datos de 347 pacientes infectados con fiebre del dengue.

Resultados: El conteo de glóbulos blancos, neutrófilos, monocitos, eosinófilos, la proporción entre neutrófilos y linfocitos, y la proporción entre monocitos y linfocitos, presentaban valores significativamente más bajos en los pacientes con fiebre del dengue al compararlos con pacientes con malaria ($P < 0,0001$). En cambio, el conteo de glóbulos rojos, el nivel de hemoglobina, hematocrito, volumen corpuscular medio, hemoglobina corpuscular media y concentración de hemoglobina corpuscular media ofrecían valores significativamente más elevados en los pacientes con fiebre del dengue que en los pacientes con malaria ($P < 0,0001$). Un modelo de árbol de decisión reveló que los valores de neutrófilos, linfocitos, concentración de hemoglobina corpuscular media y género permitían distinguir entre la infección de dengue y malaria.

Conclusión: Este estudio ha concluido que los diversos parámetros hematológicos tenían diferente relevancia para el diagnóstico de fiebre del dengue y malaria. Un modelo de árbol de decisión reveló que los valores de neutrófilos, linfocitos, concentración de hemoglobina corpuscular media y género permitían distinguir entre la infección de dengue y malaria. Además la utilización de estos marcadores nos conducirá en fase temprana a la detección, el diagnóstico y el tratamiento adecuado de estas enfermedades tropicales.

Translated from English version into Spanish by SergioLorenzi, through
